# An ontology design for validating childhood cancer registry data

**DOI:** 10.3389/fonc.2023.1212434

**Published:** 2023-07-17

**Authors:** Nicholas Nicholson, Francesco Giusti, Carmen Martos

**Affiliations:** ^1^ European Commission, Joint Research Centre (JRC), Ispra, Italy; ^2^ Belgian Cancer Registry, Brussels, Belgium; ^3^ Rare Diseases Research Unit, Foundation for the Promotion of Health and Biomedical Research in the Valencian Region (FISABIO), Valencia, Spain

**Keywords:** ontology design, description logic, data validation, data harmonization, cancer registration, childhood cancer

## Abstract

Ontologies can provide a valuable role in the work of cancer registration, particularly as a tool for managing and navigating the various classification systems and coding rules. Further advantages accrue from the ability to formalise the coding rule base using description logics and thereby benefit from the associated automatic reasoning functionality. Drawing from earlier work that showed the viability of applying ontologies in the data validation tasks of cancer registries, an ontology was created using a modular approach to handle the specific checks for childhood cancers. The ontology was able to handle successfully the various inter-variable checks using the axiomatic constructs of the web ontology language. Application of an ontological approach for data validation can greatly simplify the maintenance of the coding rules and facilitate the federation of any centralised validation process to the local level. It also provides an improved means of visualising the rule interdependencies from different perspectives. Performance of the automatic reasoning process can be a limiting issue for very large datasets and will be a focus for future work. Results are provided showing how the ontology is able to validate cancer case records typical for childhood tumours.

## Introduction

A centralised process currently exists for collecting and validating data from the European cancer registries prior to the derivation of indicators that frame the information available on the European Cancer Information System (ECIS) website (1). Dedicated software is used for the validation task, the development of which is a labour-intensive process requiring frequent interactions between the development team and the domain experts. If the rules are updated, there is significant maintenance effort to refactor the code and release the new version. The centralised data collection process is itself facing increased challenges with stricter data-privacy rules and measures, especially for data related to minors. Both these issues impinge directly on the timely availability of cancer-burden indicators which in turn compromises their value in influencing policy-related actions. Computer ontologies provide a key for the provision of more efficient and verifiable data validation processes as well as for the eventual federation of the processes to the local cancer registry level.

Ontologies are also a valuable tool in general for supporting the work of cancer registries. They provide a knowledge base able to describe entities and their relationships and consequently afford the means of capturing the semantics associated with any given domain. Moreover, the entities defined in one ontology can be linked to entities defined in another ontology. One immediate advantage is that the categorisation and linkage of entities can be made available in one application without the need of having to consult a wide set of different coding and classification standards; all the information is readily accessible. A further interesting feature is that the representation of knowledge in an ontology can be described formally using description logics (DLs). DLs constitute a branch of logics, with most DLs being decidable fragments of first-order logic (2). DLs also provide the possibility for some level of deductive reasoning and this is a useful feature for data validation, which is an essential task of cancer registries.

In order to ensure the necessary harmonisation of data-validation practices in Europe, the European Network of Cancer Registries (ENCR) agrees the rules that constrain the values and ranges cancer data variables can take. Many of these rules have multivariable dependencies and it is difficult to express them in unambiguous terms. Encoding the rules in an ontology allows them to be expressed in a formal sense *via* DL and can highlight inconsistencies in the rules that might otherwise have gone undetected (3).

The ease with which classes and their relationships can be created in an ontology editor such as Protégé (4) belies the difficulties of achieving a good ontology design. There are many ways in which the axioms can be constructed and the way in which they are formulated can have far-reaching implications on computational performance (especially where automatic reasoning is required) and on the ease of extracting information from the knowledge base. Guidelines, tools, and patterns are not widely available and ontology engineering is an emerging field. A key design principle is to achieve wide applicability of an ontology within the domain to avoid a multiplication of ontologies that cannot easily be integrated. This principle has been a driving factor in the design of the ontology for validating childhood cancer registry data.

An additional design aspect that has also to be kept in mind relates to the division of an ontology between pre and post coordination concepts. In pre coordination, knowledge about entities and their relations is asserted *a priori* in the ontology, whereas in post coordination (5), other relationships are inferred following an automatic reasoning process. Both mechanisms are useful and the degree to which one or other is used depends largely on the requirements of the application. Using a predominantly pre-coordinated ontology design would require an unmanageable set of axioms for the validation of cancer registry data. However, post coordination requires automated reasoning to make inferences based on the asserted axioms and can be computationally intensive depending on the expressivity of the DL in which the axioms are formulated.

The design of an ontology is here presented that can model the rules for validating childhood cancers. The ontology serves as the basis for developing a simple programme interface for the systematic validation of cancer registry records. The concept is notably different from the traditional approach of developing dedicated validation software. The validation conditions and machine intelligence are maintained within the ontology itself and the task of any programme interface is reduced solely to managing the data input process, invoking the standard machine reasoning tools and managing the output process. The ontology thereby provides a standalone resource that can be used for many different purposes resulting from its underlying knowledge base and can serve to reduce considerably software development and maintenance costs.

## Method

**Figure 1:**
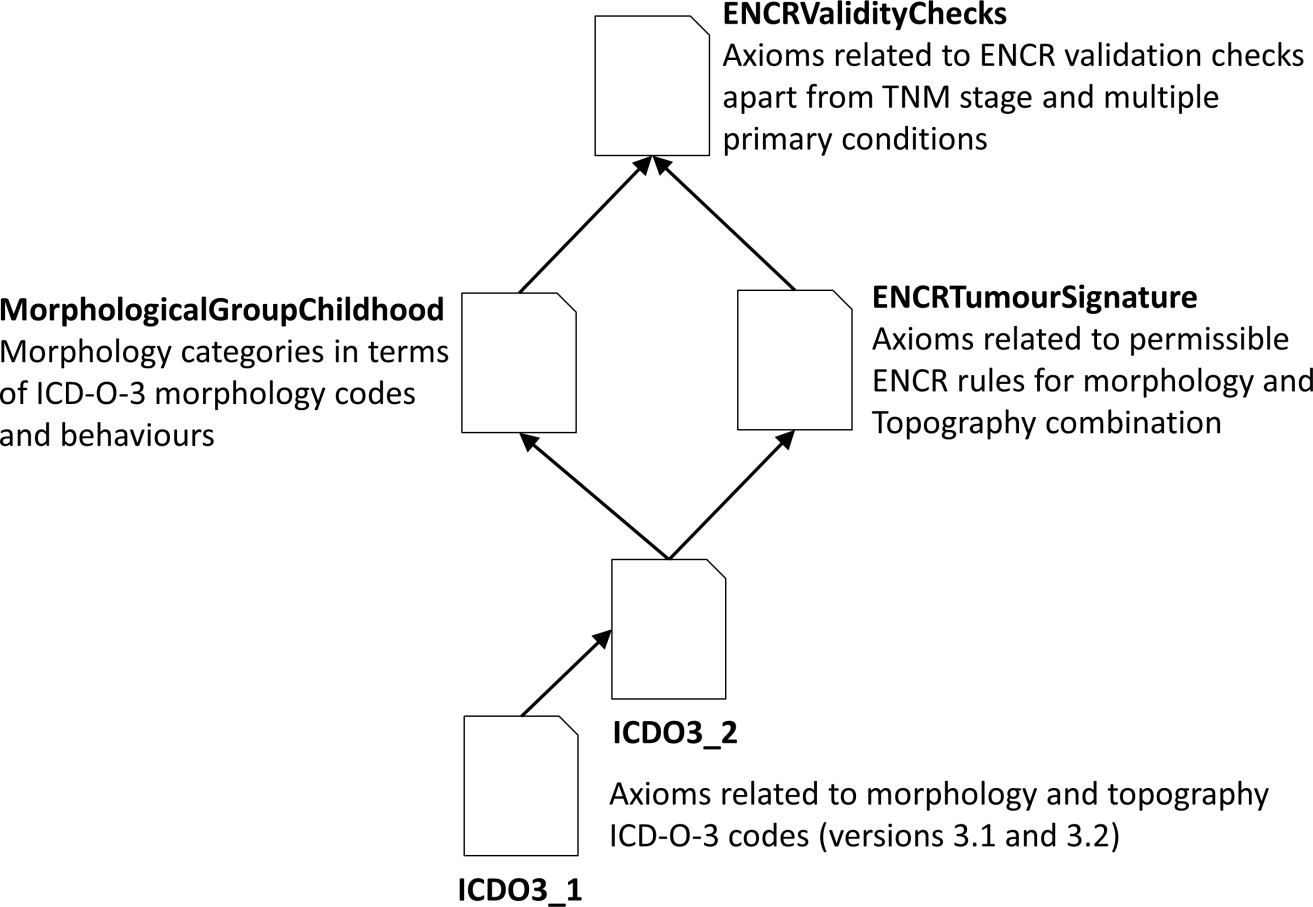
The ontology structure of the ENCR validation checks for childhood cancers, not including the cancer stage or multiple-primary checks.

An earlier tentative approach (6) showed the viability of using an ontology for validating cancer registry data and the associated advantages of expressing the rules in DL. The ontology had to be redesigned to allow a more scalable and comprehensive approach to the rules and to build on a number of shared core ontologies. Two validation modules dealing with cancer stage (7) and multiple primary tumours (8) have been developed according to this principle. Both these ontologies were developed as stand-alone applications since they are computationally quite demanding tasks and generally apply only to a subset of cancer registry case records, but they draw on the same shared core ontologies. The third application suite addressed in this article concerns the remainder of the ENCR validation checks, namely those relating to age constraints, tumour signatures, basis of diagnosis, grade, and sex. **Figure 1** illustrates the ontology structure, in which the international classification of diseases for oncology, third edition, first revision (ICD-O-3.1) and the international classification of diseases for oncology, third edition, second revision (ICD-O-3.2) modules contain all the ICD-O third edition codes (ICD-O-3) and updates. The MorphologicalGroupChildhood ontology can be swapped out relatively seamlessly dependent on the requirements of the application. It has been designed for validating childhood cancer data which forms the focus of this article and draws from the grouping and subgrouping of the ICD-O-3 codes defined by the international classification of childhood cancer, third edition (ICCC-3) update 2017 (9). This module however can be replaced by any other grouping of ICD-O-3 codes and the resulting application used also for validating adult cancer records.

The ENCRTumourSignature ontology provides the permissible code couplets for topography and morphology values according to the ENCR rules (10) and can itself be used also as a standalone ontology if required. The modular approach to creating ontologies *via* the mechanism to import ontologies into other ontologies is of great benefit to the scalability, reuse and maintenance of ontologies.

Computational performance accounts for one of the main current drawbacks to automatic reasoning in DL and requires further care in how the ontology is designed. DLs are classified by their expressivities, where expressivity describes the types of operators permitted. Higher expressivities are computationally more demanding but allow more complex reasoning. For example, the DL expressivity ELH (existential language with role hierarchy, allowing concept intersection, existential restrictions, and sub-properties) on which SNOMED CT (11) is modelled, can classify an ontology subsumption hierarchy in polynomial time (PTIME) (12). The higher expressivity of SHIQ has a worst-case complexity of EXPTIME (13) and SROIQ of N2EXPTIME (14). Whereas the introduction of optimised implementations of Tableau-based algorithms has enabled use of higher expressivities in practical applications even for complexities higher than PTIME (13), care has to be exercised to limit the expressivity as far as possible, especially with applications involving many thousands of axioms.

The ENCRValidityChecks ontology includes axioms relating to the constraints on morphology/topography combinations (or tumour signatures), basis of diagnosis, sex, grade, and age at diagnosis or incidence date. The tumour signature axioms (defined in the ENCRTumourSignature ontology) verify that the topography and morphology codes for each cancer case accord with the combinations considered permissible by the ENCR rules. The structure of the tumour signature ontology module passed through a number of design attempts to find an acceptable compromise between usefulness and efficiency. A major issue related to the very large number of morphology codes specified by ICD-O-3 (just under two thousand) and the combination of these codes with a substantial number of topography codes (330 codes).

In an initial design we subclassed the topography codes from the morphology codes, but this forced a coupling in the classification trees between morphologies and topographies. In other ontology modules we needed to specify existential relationships with morphology without automatically pulling in the associated topographies. Nor would it have helped to subclass the morphology codes from the topography codes since this would have resulted in the same problem when specifying existential relationships with topography. Moreover, the open world assumption of DLs meant that we were unable to specify the necessary class subsumption axioms required for automatic validation of the permitted morphology and topography combinations for a given tumour signature. Whereas this caused no difficulty in visualising the asserted topography-morphology relationships in the ontology’s graphical user interface, it did mean that such information could not be inferred by the reasoner and therefore not optimal from the point of view of automating the validation checks themselves.

**Figure 2:**
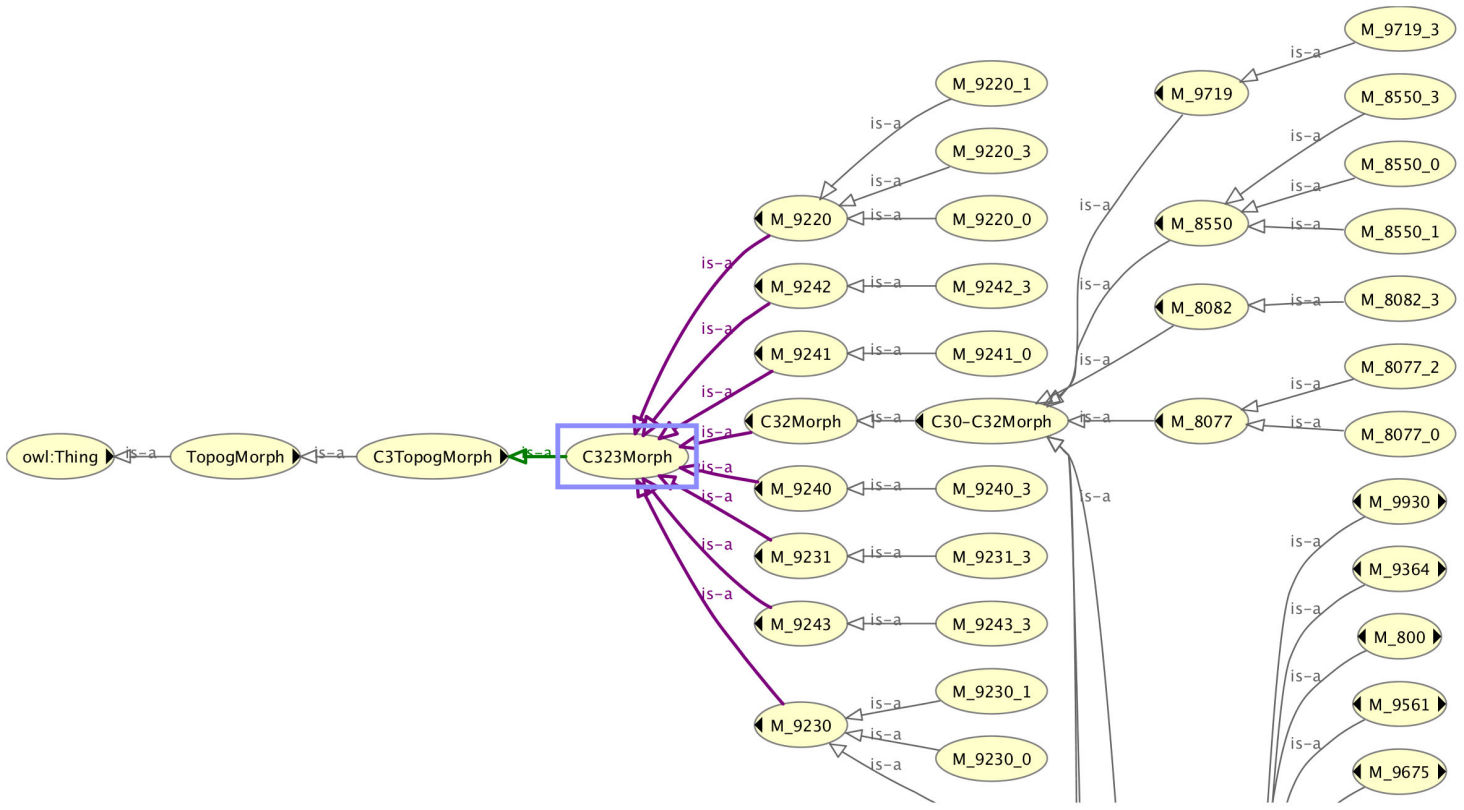
Part of the classification tree of the dummy topography code class C323Morph showing the associated morphologies.

To overcome these issues, we had little option other than to duplicate the entire topography classification hierarchy (under a dummy name) and subclass the morphology codes under the dummy topography classification tree. This allowed a decoupling of the “real” topography codes from the morphology codes (since the morphology codes were then only associated with the dummy topography codes). Given that the real topography codes can be determined from the similarly named dummy topography codes, it is still possible from the graphical user interface to see which morphology codes are associated with a given topography code (and vice versa). This may be appreciated from the partial classification tree of the dummy topography code called “C323Morph” in **Figure 2**, where it is clear from the name that the associated real topography code is C323. All the morphologies associated with this code are visible in the classification tree under the dummy topography class.

**Figure 3:**
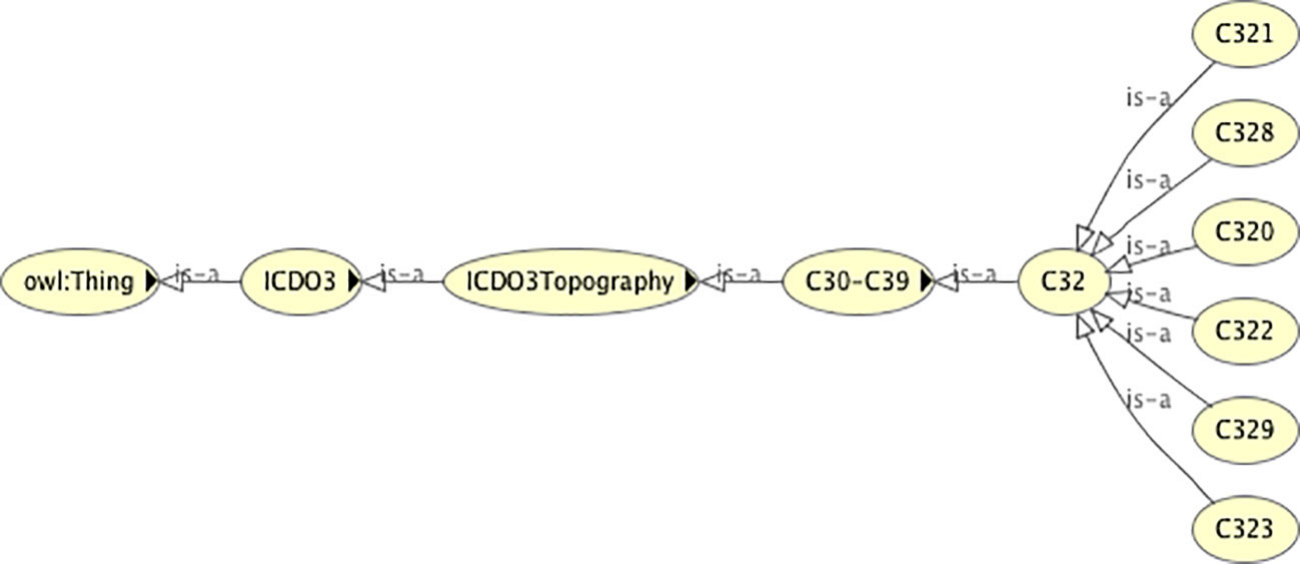
The classification tree of the real topography code class C323, showing its position in relation to the associated three-digit topography code C32.

Apart from the association of the morphology codes, there is one slight difference between the classification trees of the dummy topography codes and the real topography codes shown for the topography code C323 in **Figure 3**. The four-digit C323 code is subclassed from its three-digit code parent C32, in contrast to the dummy C323 code (C323Morph) that is the superclass of the three-digit dummy topography code C32Morph. The reasons for inverting the classification tree for the dummy code are firstly to avoid unnecessary duplication of the morphology codes under the dummy topography codes, and secondly to ensure that the existential relationships acting on the morphology codes are correctly specified. The four-digit dummy topography codes have more morphologies associated with them than the three-digit codes and specifying the three-digit codes should not pull in the morphology codes that are only associated with the more granular four-digit codes.

**Figure 4:**
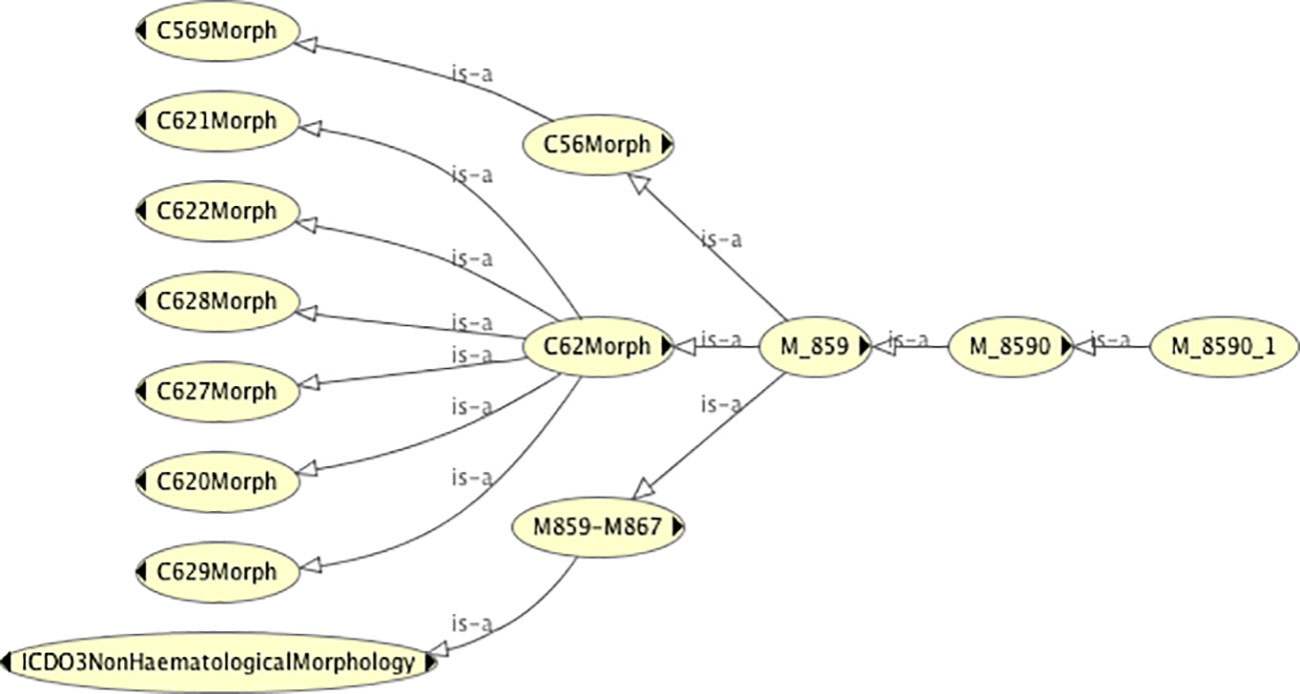
The classification tree of the morphology code class 8590/1, showing the dummy topography codes with which it is associated.

Ascertaining the dummy topography codes (and therefore the real topography codes) with which a given morphology is associated is also straightforward. **Figure 4** shows the topography codes associated with the morphology code 8590/1 (namely C56 and C62).

The class subsumption axioms for a valid tumour signature can then be defined along the lines:


∃hasMorphology(C000Morph)⊓∃hasTopography(C000)⊏VALID_TumourSignature

which states that the conjunction (⊓) of an existential relationship (∃) of the topography code C000 and an existential relationship of the dummy topography code C000Morph (under which all the permitted morphologies for the topography code C000 are defined) is a valid tumour signature. This axiom clearly has to be duplicated for all topography codes and results in any valid combination of morphology and topography being subsumed under the class VALID_TumourSignature, allowing a simple test in a batch programme for validating compliant cancer case records.

The checks for basis of diagnosis and grade also raised an interesting challenge for handling them in description logic. DLs incorporate monotonic logic, meaning that a conclusion cannot vary with the addition of a new set of premises. In practical terms, this means that default values or exceptions cannot be attributed and cannot therefore be used to model the scenario in which a rule takes a default value that may then be overridden for a given condition. The rule tables for both basis of diagnosis and grade are in fact expressed in terms of exceptions to default values.

In order to circumvent this limitation we needed to introduce a default test value (for a given rule) and a violation flag. The default test value is defined either as default-valid or default-invalid. In the case of a default-valid test, if a given set of values violate the rule, then an invalid condition is flagged and vice versa. Thus, the user/application analysing the test results would need to look for any associated violation flag. In the absence of a violation flag, it can be concluded that the test result is valid or invalid depending on the default test value. An example of an axiom providing a default-invalid test value for the basis of diagnosis corresponding to *clinical investigation* is:


∃prevalidatedBasisOfDiagnosis(BoDcode2_Investigation)⊏InvalidBoDDefaultCase

which states that any specified basis of diagnosis code 2 (clinical investigation) is an invalid basis of diagnosis default case. A rule for overriding this default value is:


$$\begin{array}{c} \exists prevalidatedBasisOfDiagnosis\left(BoDcode2\_Investigation\right)⊓\\ \exists hasMorphology\left(M\_8960\_3\right)⊓\\ \exists hasTopography\left(C64\right)⊏VALID\_BoD \end{array}$$

Which, for a specified basis of diagnosis code 2, a morphology code 8960/3 and a topography code C64 (and all its four-digit subclasses), renders the check valid.

The axioms for validating age at diagnosis are less convoluted since they only require verification against minimum and maximum values. For combinations of topography and morphology that have an age restriction, the axioms for a minimum age limit take the form:


∃hasMorphology(M801−M804)⊓∃hasTopography(C15) ⊏∃expectedAge(>14)

which states that the conjunction of morphology codes 801-804 (and all the associated subclasses) with topography code C15 (and associated subclasses) have an expected age greater than 14 years. The axioms for deriving the validation at post-coordination take the form:


∃expectedAge(> 14)⊓∃patientAgeAtDiagnosis(<15)⊏WARNING_age

for which a specified patient age at diagnosis less than 15 years when the expected age is greater than 14 years generates a warning condition.

The axioms for validating sex are simple, since they involve only a test on topography Thus:


∃hasTopography(C60−C63)⊏∃IsSexOf(Male)

ensures that topography codes C60-C63 are associated with the male sex, with the validation rule:


∃IsSexOf(Male)⊓∃prevalidatedSex(Female)⊏InvalidSexCombination

that states if the specified parameters require a male sex and a female sex is specified, then the cancer case will be subsumed under the class InvalidSexCombination.

## Results

**Figure 5:**
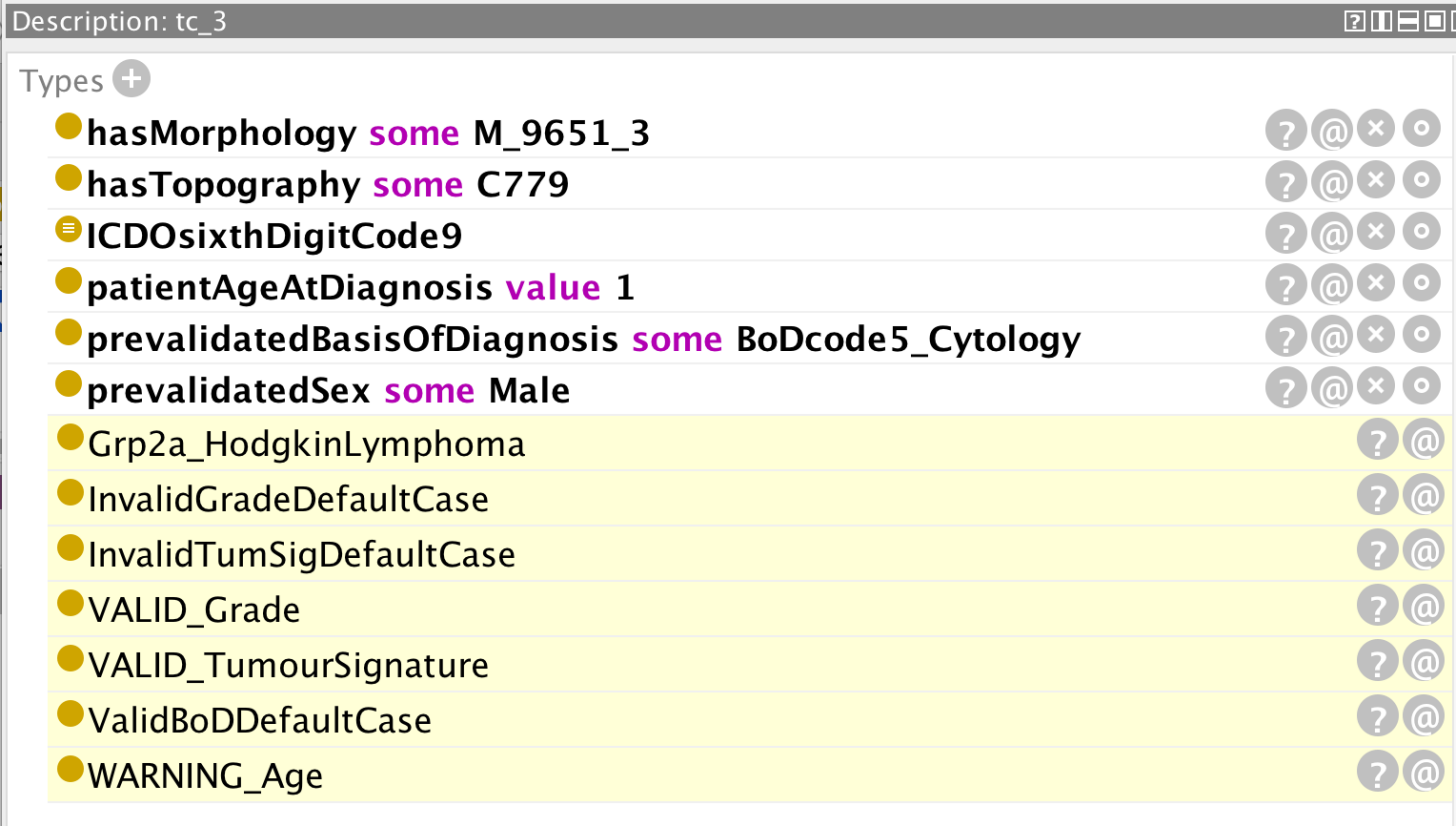
Inferences (highlighted lines) drawn by the reasoner on the basis of the specified parameters (non-highlighted lines). The values of grade, basis of diagnosis, and tumour signature are all valid but the reasoner has flagged a warning for the specified age.

**Figure 6:**
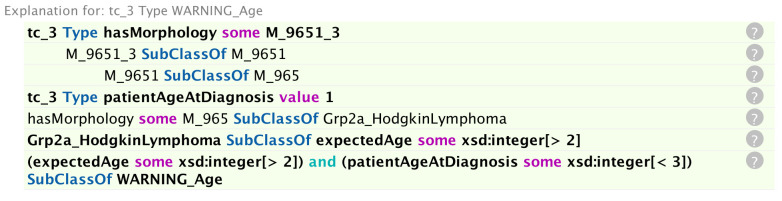
Explanation of why the reasoner generated a warning on the age specified in **Figure 5**.

**Figure 7:**
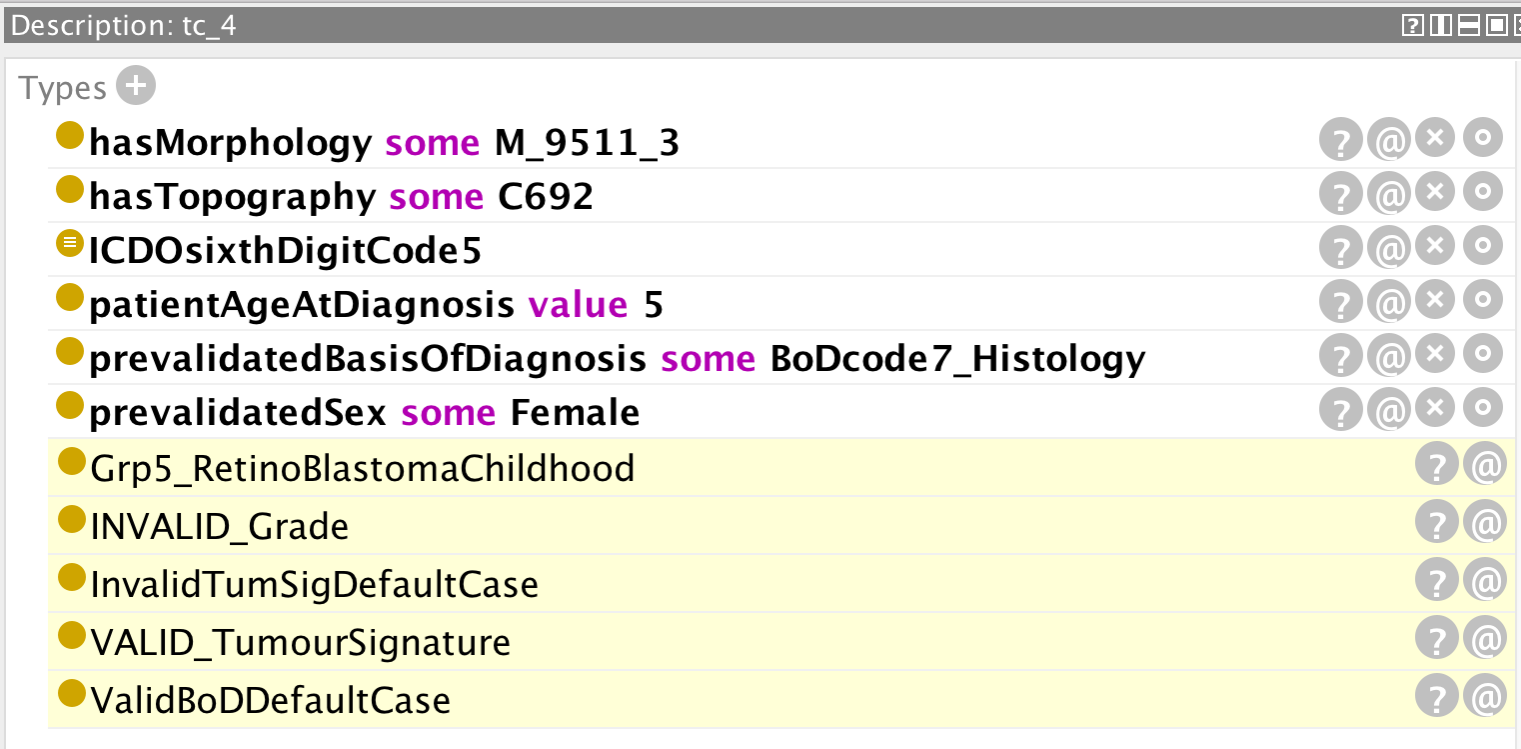
Inferences (highlighted lines) drawn by the reasoner on the basis of the specified parameters (non-highlighted lines). The reasoner has inferred an invalid grade on the basis of the specified morphology.

**Figure 8:**
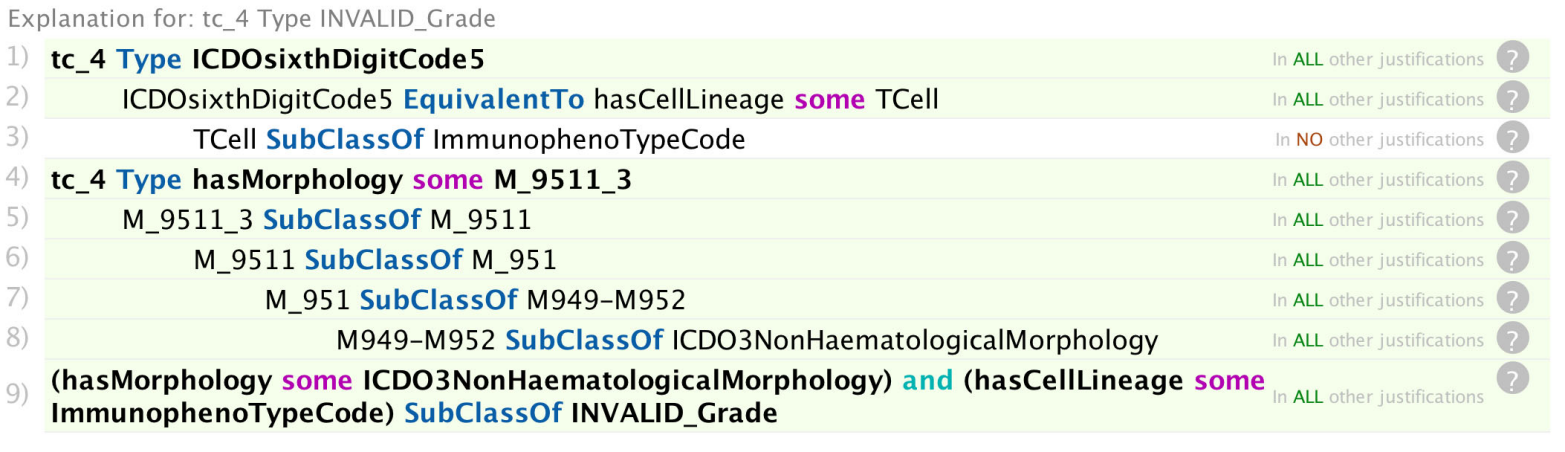
Explanation of why the reasoner inferred an invalid value of the grade specified in **Figure 7**.

**Figure 9:**
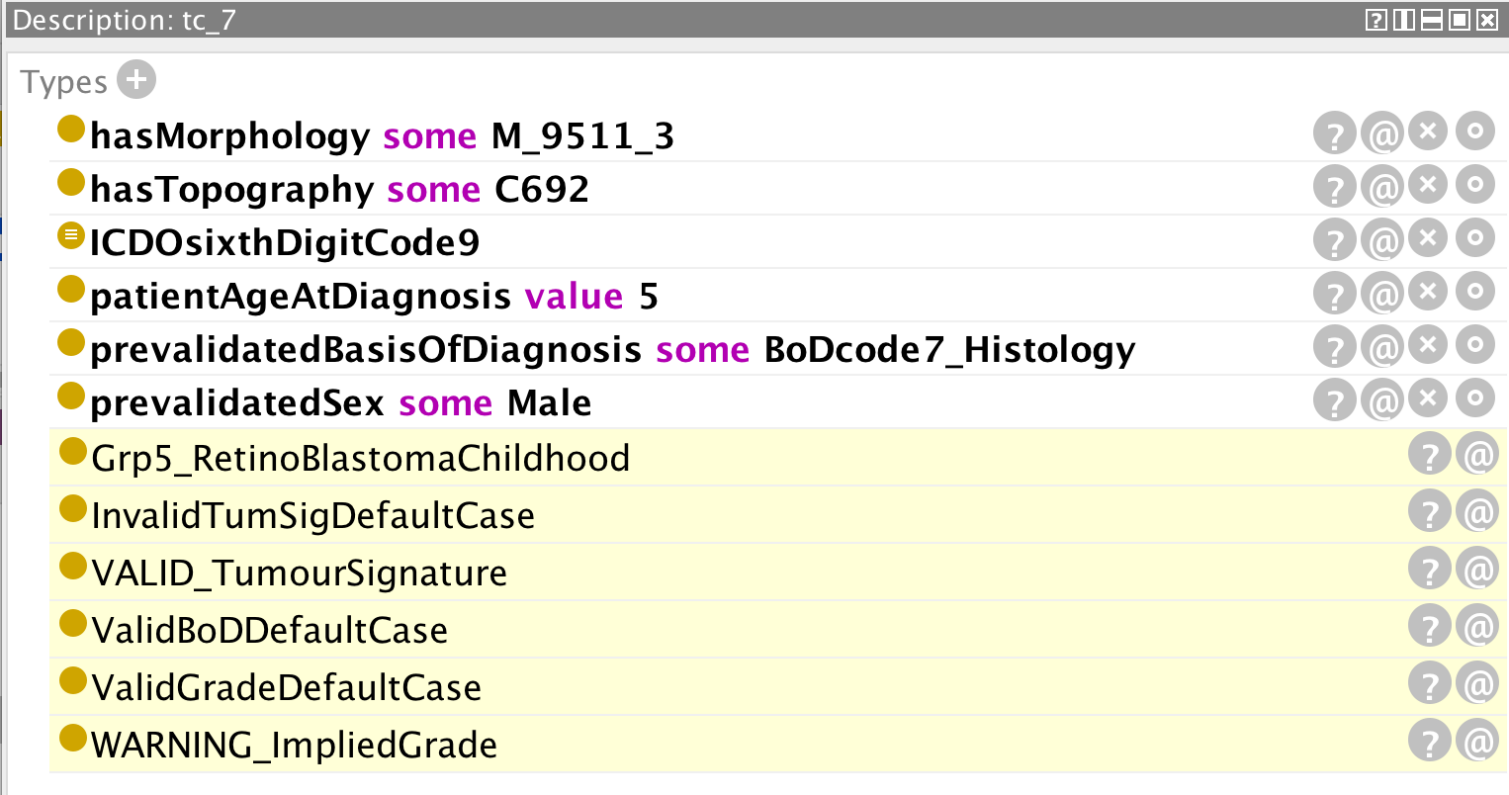
Inferences (highlighted lines) drawn by the reasoner on the basis of the specified parameters (non-highlighted lines). The reasoner has inferred an implied grade on the basis of the specified morphology.

**Figure 10:**
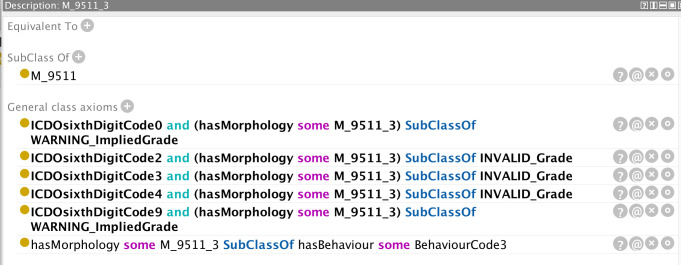
Class definition of the morphology code 9511 from which the implied value of grade can be determined.

**Figure 11:**
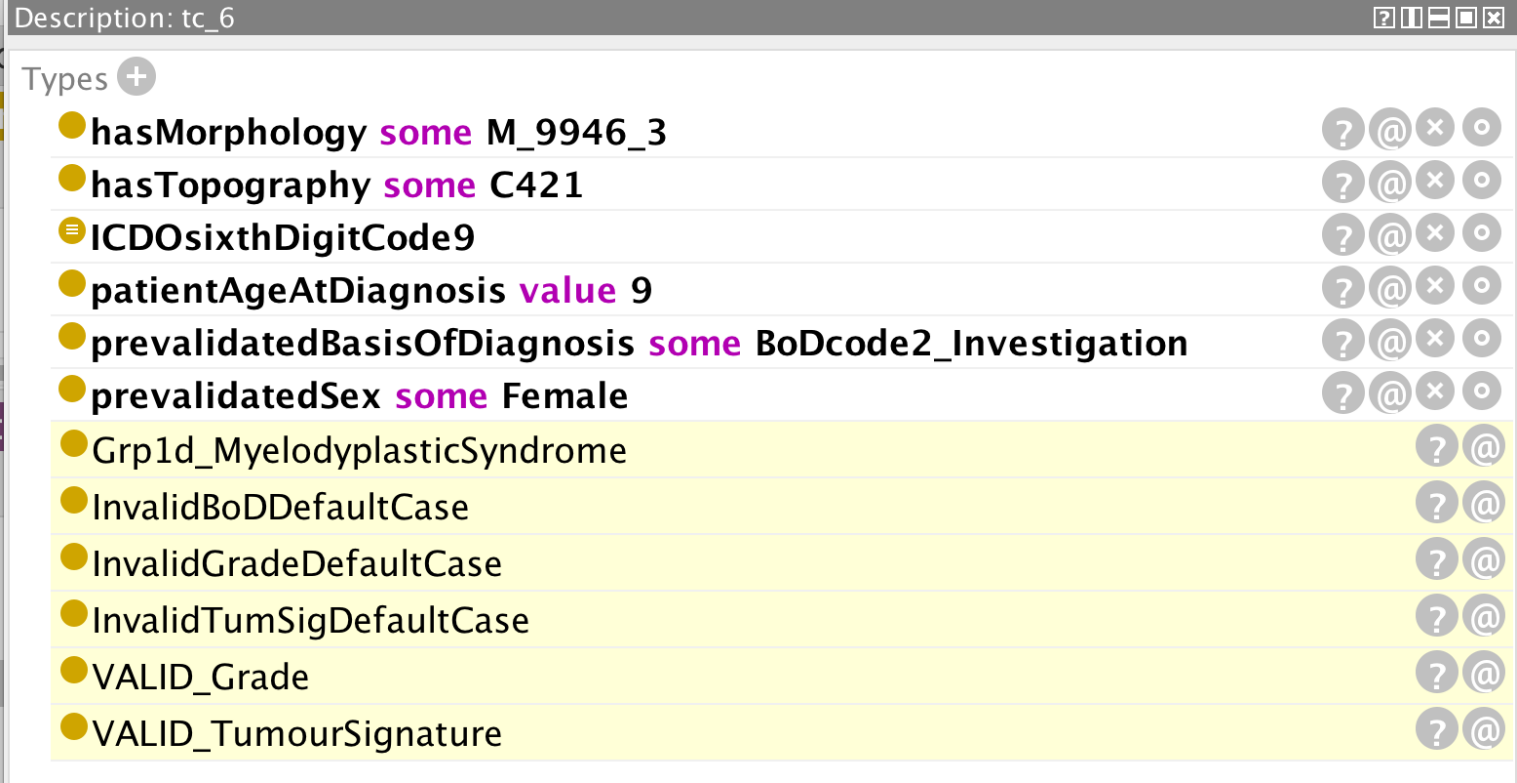
Example of a cancer case with an incorrect basis of diagnosis code (code 2, describing a non-microscopic clinical investigation) which is not admissible for this morphology type.

**Figure 12:**
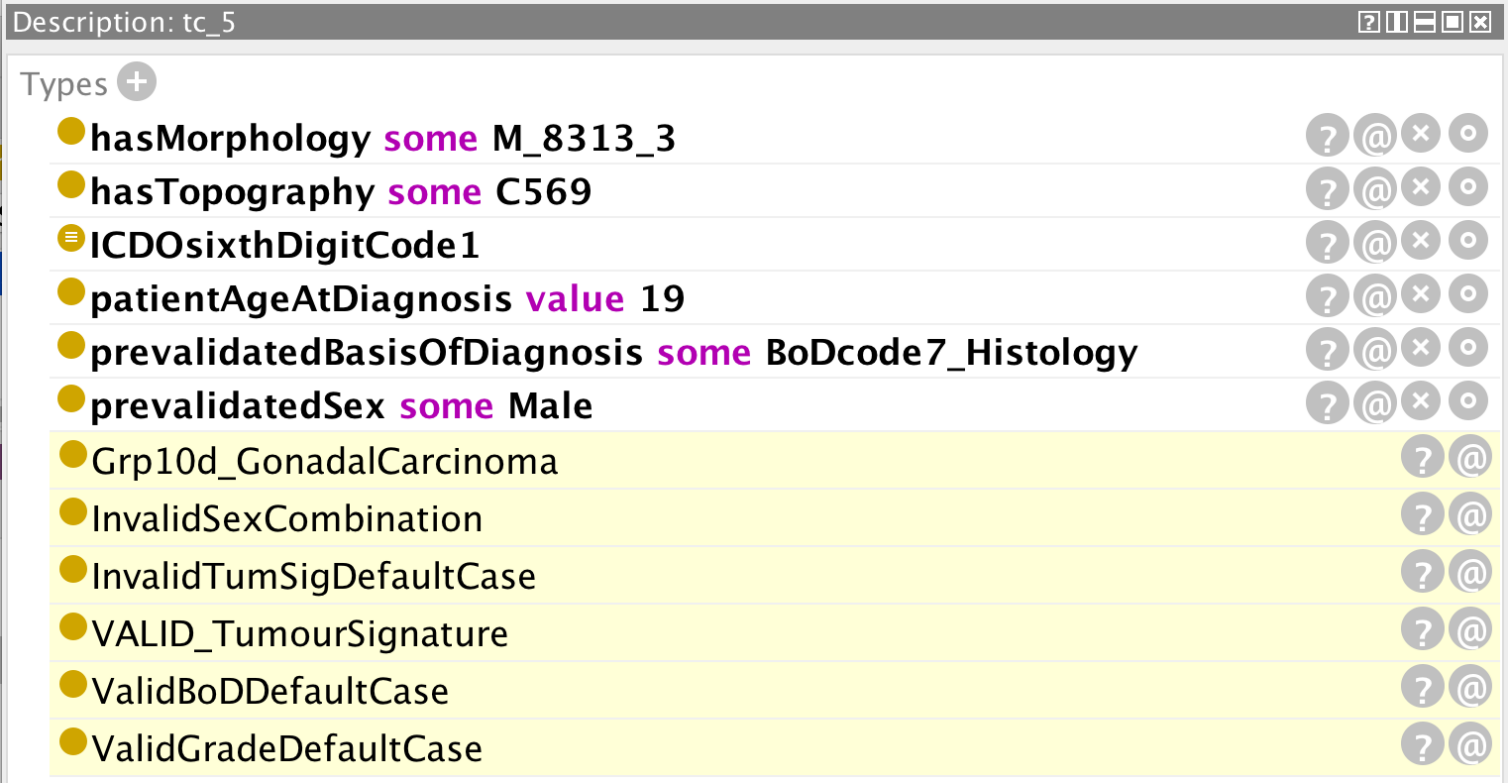
Example of a cancer case with an error in the encoding of the patient’s sex.

Examples are provided in **Figures 5**–**12** of how the ontology handles the ENCR data-validation requirements *via* the post-coordination mechanism for a number of imaginary cancer-case scenarios. The yellow highlighted lines in the figures refer to the inferences made by the reasoner on the basis of the information passed to it (represented by the non-highlighted lines).

In **Figure 5**, the pre-specified parameters are: morphology 9651 and behaviour (code 3 signifying malignancy in the primary site) – the composite code 9651/3 signifying Hodgkin lymphoma; patient age at diagnosis (one-year old); basis of diagnosis (code 5 signifying cytology); sex (male); and an ICD-O sixth digit code of 9. The ICD-O sixth digit code (grade of the tumour) can take the code values 0-9 and is used for histologic grading or differentiation. Codes 1–4 are only used for non-haematological or solid tumours (with the exception of morphology 9801), and codes 5–8 only for haematological tumours. Code 0 (not applicable) or code 9 (unknown) can be used for both classes of tumour. The highlighted yellow lines in the figure represent the inferences made by the reasoner on the basis of the pre-specified parameters. It can be seen from these inferences that the given parameters constitute invalid default cases for both tumour signature and grade, but that these default cases have been overridden by the respective “VALID” flags. Conversely, a default valid basis of diagnosis has been inferred and since this has not been overridden by an “INVALID” flag, it can be assumed that the basis of diagnosis is also valid. In addition, the reasoner has inferred an unlikely age for the input age parameter. The rationale for the inference of any given statement can be ascertained by clicking on the question mark next to the inferred statement. The explanation for the age warning (**Figure 6**) is that the expected age at diagnosis is greater than 2 for Hodgkin lymphomas (classified under the ICCC-3 group IIa, which the reasoner has deduced from the morphology code).

In **Figure 7**, the reasoner has inferred an ICCC-3 group V morphology (retinoblastoma) and an invalid grade code. The error results from the attempt to ascribe an immunophenotype grade code (codes 5–8) to a non-haematological tumour (**Figure 8**). Since this is an absolute rule that is triggered for all non-haematological morphologies (c.f. line 9 of **Figure 8**), there is no valid grade default case in this instance.

**Figure 9** distinguishes between an invalid grade code inference and a grade code warning. Certain morphologies have an implied grade and these codes should be used instead of leaving the value unspecified (grade code 9). In order to determine the implied value(s) of a grade code, the reasoner is less informative and it is necessary to access the class description of the relevant morphology code (in this case 9511, c.f. **Figure 10**). The only grade code that is not invalid for this morphology (which is a non-haematological morphology) is 1 and thus it can be inferred that the implied grade is 1. Extra classes and rules could be added to the ontology to provide the implied grades directly but this is one of the compromises taken to avoid affecting performance further. An application programme interfacing with the ontology could determine the implied grade as easily as the user on the basis of the asserted axioms.

**Figure 11** is an example of a cancer case with an erroneous basis of diagnosis code (non-microscopic clinical investigation) which is not admissible for this morphology type (juvenile myelomonocytic leukaemia). The rules for a basis of diagnosis code 2 are by default invalid, and valid cases are flagged as exceptions. The grade and tumour signature combinations also derive from invalid default conditions but these have been overridden by the valid flags (ultimate 2 lines of **Figure 11**).

**Figure 12** is an example of a cancer case with an error in the encoding of the patient’s sex. Topography code C569 (ovary) pertains solely to the female sex and the reasoner has inferred the error correctly,

Reasoning times are dependent on the specific reasoner used as may be appreciated from **Table 1** which shows the time to classify the ENCR validation check ontology with and without the ENCR tumour signature checks for three reasoners (FaCT++, Hermit, and Pellet) on a 3GHz Intel Core i7 processor with 16 GB RAM. It is interesting to compare the performance of the Hermit reasoner with the other two reasoners in relation to the ENCR tumour signature ontology. It is not immediately clear why Hermit should take significantly longer to classify this particular ontology than the other reasoners (especially since they all use optimised Tableau-based techniques).

**Table 1:** Summary of the expressivities and size of the various ontologies comprising the ENCR validation application, with comparison of reasoning performance between various reasoners.

Ontology	DL Expressivity	No. logical axioms	GCI count	Reasoner	Execution time (seconds)
ENCR validation (including ENCR tumour signature)	ALC^(D)^	14,138	7,769	FaCT++	7
				Hermit	10
				Pellet	5
ENCR validation (excluding ENCR tumour signature)	ALC^(D)^	10, 418	7,439	FaCT++	5
				Hermit	1
				Pellet	2.5
ENCR tumour signature (including the ICD-O-3 ontologies)	ALC	7,699	1,534	FaCT++	2
				Hermit	9
				Pellet	2

The GCI count refers to the number of general concept inclusion axioms, which Protégé defines as axioms whose subclass is a complex class expression (and more demanding in terms of reasoning). The DL expressivity ALC denotes attributive language (AL) with complex concept negation (C). The superscript (D) relates to the use of datatype properties..

In terms of the domain knowledge encapsulated in the ontology, **Table 2** shows a breakdown of the knowledge that can be ascertained *via* the pre-coordination or post-coordination processes. Pre-coordinated knowledge remains accessible also after post coordination.

**Table 2:** Summary of the type of information derivable from the knowledge base *via* the pre and post coordination mechanisms.

Item of knowledge	Mechanism for deriving the knowledge
All the codes for: ICD-O-3 (morphology, topography, behaviour, grade); basis of diagnosis; morphology groups	Pre coordination
Division of morphologies between haematological and non-haematological	Pre coordination
Permissible values of morphology for a given value of topography	Pre coordination (c.f. **Figure 2**)
Permissible values of topography for a given value of morphology	Pre coordination (c.f. **Figure 4**)
Morphologies with a specific given behaviour	Pre coordination
Topographies associated with male/female sex	Pre coordination
Whether a given morphology and topography code is a valid combination for a given basis of diagnosis	Post coordination
Whether a given age of diagnosis is unlikely for a given morphology or combination of morphology and topography	Post coordination (c.f. **Figure 5**)
Whether a given topography and morphology combination is a valid tumour signature	Post coordination (c.f. **Figure 5**)
The morphology group to which a topography and morphology combination is associated	Post coordination (c.f. **Figure 5**)
Whether a given grade is valid/invalid for a specific morphology or combination of morphology and topography	Post coordination (c.f. **Figure 7**)
Whether a morphology has an implied grade	Post coordination (c.f. **Figure 9**)

**Figure 13:**
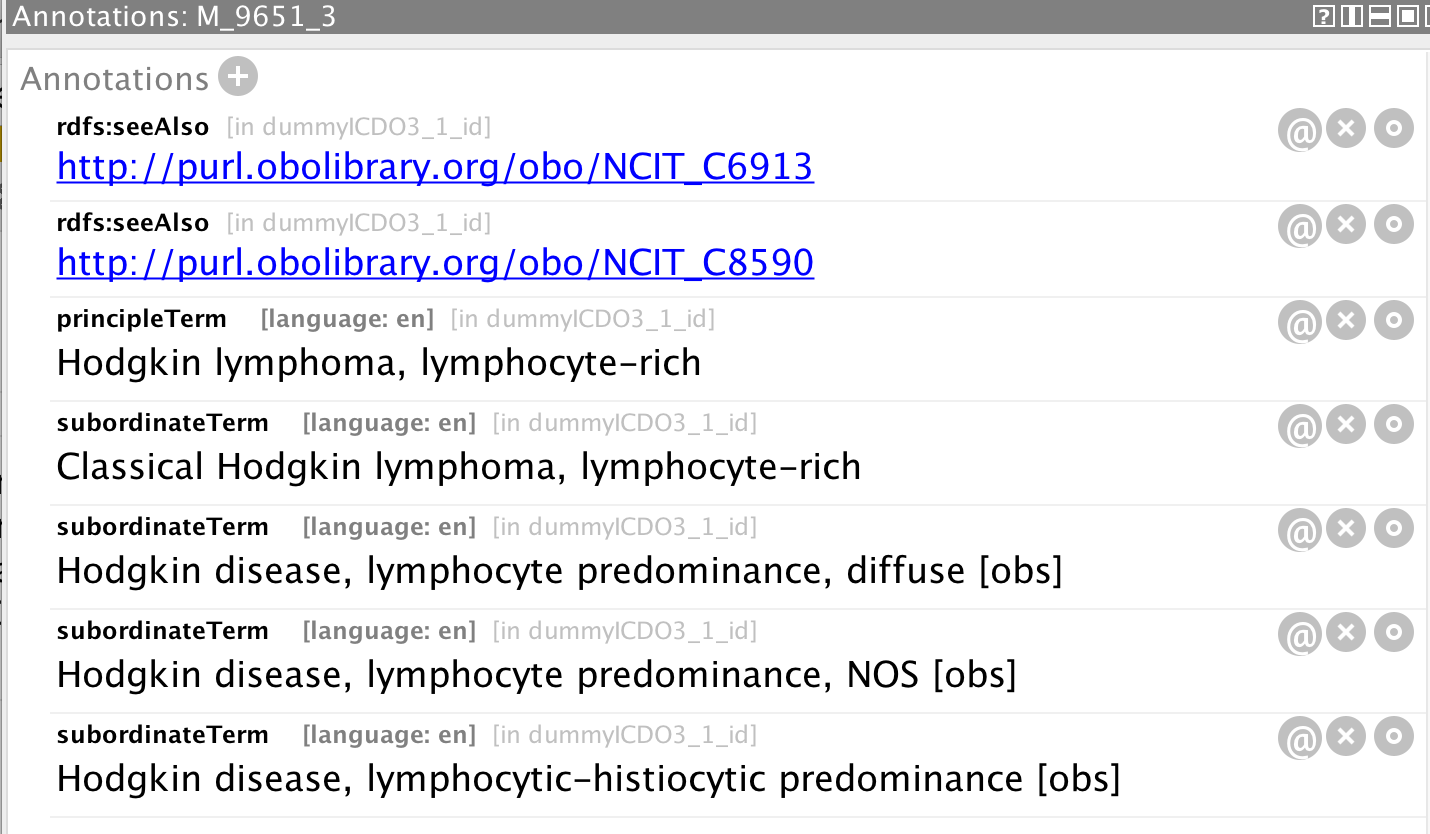
Annotations associated with the morphology class M_9651_3 representing the ICD-O-3 morphology code 9651/3.

A further useful functionality of ontologies comes from the ease of annotating a class with other information. **Figure 13** shows the annotations associated with the ICD-O-3 morphology code 9651/3, from which it can be seen that all the descriptive text of ICD-O-3 can be captured for a given entity, as well as links to other resources (such as on-line data dictionaries, thesauri, and other ontologies). This allows access to a comprehensive set of knowledge describing the resource directly from a single application.

## Discussion

The ontology described here for validating childhood cancer registry cases is a novel alternative approach for data cleaning processes that have traditionally been performed *via* dedicated application software. Using ontologies for these tasks brings a number of advantages. One key strength concerns the use of DL to describe the data validation rules in a formal sense. Formalising the rules not only removes the inherent ambiguity of specifying them in natural language but can also detect inconsistencies within them. A further benefit of DL relates to its amenability to automatic machine reasoning tools that can pre-empt the need to handle complex validation conditions in dedicated software. Keeping the intelligence within the ontology allows a simpler programme interface and reduces software development and maintenance costs.

Ontologies also permit the integration of the data rules with the code classification systems. Cancer registries have to deal with many hundreds of codes from a variety of classification standards and from this standpoint alone, ontologies can structure the information to make it much more readily accessible. By expressing entities and relations in a comprehensive knowledge base, the task of ascertaining and verifying codes and their dependencies becomes a relatively straightforward task. This way of structuring information makes it considerably easier to verify data validation rules that otherwise require multiple table look-ups and also greatly facilitates maintenance issues by keeping the codes, rules, and variable values in a single application. An important corollary to this is the default functionality of OWL ontologies to maintain persistent metadata links *via* the international resource identifiers (IRIs) they assign to each entity as well as their ability to link to other metadata contexts. Access to a relevant set of comprehensive metadata is of fundamental importance to secondary data usage where data users need to understand the meaning of the data. For example, the cancer sites displayed on the ECIS data browser consist of groups of individual topography codes. Ontologies encode this information directly and moreover allow linkage *via* linked open data (LOD) principles to other metadata resources, such as thesauri and data dictionaries. Data users therefore have access *via* a single entry point to a wide source of information and reference material that extends far beyond the immediate classification needs of the ontology itself.

Furthermore, unifying the validation checks with the code classification systems ensures synchronisation of code classification editions with the data-validation rule base and a more thorough versioning control than can be assured *via* distributed software. These aspects are critical to expediting the devolution of centralised data cleaning process to the local registry level and also to facilitating any eventual audit process for formally ensuring consistency of local data-cleaning processes.

An additional motivation that may perhaps be the most far-reaching is the potential stimulation of wider collaboration and development within the pan European cancer registry domain. It can justifiably be argued that code classification systems have been structured without the wider contexts in mind and lead to hierarchies that are not optimal to implementation in software. The way in which we had to group the morphology codes in the ontology design under many different class hierarchies dependent upon the particular rules points to the need for a more optimal code classification. This incidentally provides a useful example to show how the logic of ontologies can feed back into improving the representation and structuring of domain knowledge. Disease registry staff with knowledge of how ontologies work would provide a key input into future formulations of such classification systems.

It has to be emphasised however that the design of an ontology is a critical factor in its usefulness and performance. A modular structure such as the one described here helps limit complexity and aids maintenance and further development. It also allows the creation of specific dedicated ontologies using a “pick and mix” approach. For example, the childhood cancer ontology can be made equally applicable to adult cancers simply by swapping out the childhood cancer morphology grouping by an adult cancer morphology grouping. Likewise the most appropriate cancer stage ontology can be used, for example TNM for adult cancers or one that models a stage system more appropriate to childhood cancers, such as described by the Toronto childhood cancer stage guidelines (15). As long as the umbrella class names remain the same, no other changes need to be made in the other ontology applications that import the morphology grouping module. A modular structure is also useful for optimising performance for a particular set of checks and for deciding which reasoner may be best to use (c.f. **Table 2**).

Performance of automatic reasoning can provide limitations in the validation of large data sets although there are a number workarounds that do not negate the usefulness of ontologies for this task. Limiting DL expressivity to EL reasoning allows algorithms to complete in polynomial time and most cancer-registry validation rules can be handled within these constraints. Where higher expressivities are required, data sets can be ingested as a series of smaller sets and improve efficiency (since reasoning time is not linearly proportional to data-set size). There is also the possibility of exploiting the strengths of the various DL reasoners and future work will seek to understand the reason behind the performance differences observed in **Table 2** in order to improve performance on the basis of the types of axioms. Whereas others have addressed comparisons of reasoners (16–22), work has generally focused on their accuracy, the types of operations and platforms they support, and overall performance rather than the strengths of the reasoners given a particular ontology structure. The OWL2Bench however provides a promising approach (23). Optimisation of reasoning processes and algorithms continues to be an active field of research.

A further consideration is that data validation is a highly parallelisable process and other semantic web tools are available for interfacing with an ontology apart from DL reasoning, such as SPARQL queries and direct access *via* a computer programme using the OWL-API application programme interface. The latter provides a solution superior to coding all the information in a dedicated computer programme. The OWL-API provides access to both pre- and post-coordinated information and where reasoning performance is a limiting factor, the computer application can swap out the reasoning functionality with its own dedicated logic on the basis of the ontology axioms without having to redefine all the rules and entity relationships. Thus, encoding domain knowledge in an ontology provides many advantages and flexibility in the way of handling information and deriving relationships beyond those explicitly expressed. For data validation purposes at least, this functionality is of considerable benefit.

## Data availability statement

The datasets presented in this study can be found in online repositories. The names of the repository/repositories and accession number(s) can be found below: The datasets generated for this study are available from: http://data.europa.eu/89h/6f69e886-e5bc-4cd8-9b2d-8aaccf836789. License: European Commission Reuse and Copyright Notice.

## Author contributions

Conceptualisation, NN; Data curation, FG, CM; Formal analysis, NN, FG, CM; Methodology, NN, FG and CM; Project administration, NN, CM; Software, NN; Supervision, CM; Validation, FG, CM; Writing—original draft, NN; Writing—review & editing, NN, FG, CM. All authors contributed to the article and approved the submitted version.
